# Maternal Diarrhea and Antibiotic Use are Associated with Increased Risk of Diarrhea among HIV-Exposed, Uninfected Infants in Kenya

**DOI:** 10.4269/ajtmh.19-0705

**Published:** 2020-02-24

**Authors:** Emily L. Deichsel, Patricia B. Pavlinac, Dorothy Mbori-Ngacha, Judd L. Walson, Elizabeth Maleche-Obimbo, Carey Farquhar, Rose Bosire, Grace C. John-Stewart

**Affiliations:** 1 Center for Vaccine Development and Global Health, University of Maryland School of Medicine, Baltimore, Maryland;; 2 Department of Global Health, University of Washington, Seattle, Washington;; 3 United Nations Children’s Fund (UNICEF), New York, New York;; 4 Department of Epidemiology, University of Washington, Seattle, Washington;; 5 Department of Pediatrics, University of Washington, Seattle, Washington;; 6 Department of Medicine, University of Washington, Seattle, Washington;; 7 Child Acute Illness and Nutrition (CHAIN) Network, Nairobi, Kenya;; 8 Department of Pediatrics and Child Health, University of Nairobi, Nairobi, Kenya;; 9 Center for Public Health Research, Kenya Medical Research Institute (KEMRI), Nairobi, Kenya

## Abstract

HIV-exposed, uninfected (HEU) children are a growing population at particularly high risk of infection-related death in whom preventing diarrhea may significantly reduce under-5 morbidity and mortality in sub-Saharan Africa. A historic cohort (1999–2002) of Kenyan HEU infants followed from birth to 12 months was used. Maternal and infant morbidity were ascertained at monthly clinic visits and unscheduled sick visits. The Andersen–Gill Cox model was used to assess maternal, environmental, and infant correlates of diarrhea, moderate-to-severe diarrhea (MSD; diarrhea with dehydration, dysentery, or related hospital admission), and prolonged/persistent diarrhea (> 7 days) in infants. HIV-exposed, uninfected infants (*n* = 373) experienced a mean 2.09 (95% CI: 1.93, 2.25) episodes of diarrhea, 0.47 (95% CI: 0.40, 0.55) episodes of MSD, and 0.34 (95% CI: 0.29, 0.42) episodes of prolonged/persistent diarrhea in their first year. Postpartum maternal diarrhea was associated with increased risk of infant diarrhea (Hazard ratio [HR]: 2.09; 95% CI: 1.43, 3.06) and MSD (HR: 2.89; 95% CI: 1.10, 7.59). Maternal antibiotic use was a risk factor for prolonged/persistent diarrhea (HR: 1.63; 95% CI: 1.04, 2.55). Infants living in households with a pit latrine were 1.44 (95% CI: 1.19, 1.74) and 1.49 (95% CI: 1.04, 2.14) times more likely to experience diarrhea and MSD, respectively, relative to those with a flush toilet. Current exclusive breastfeeding was protective against MSD (HR: 0.30; 95% CI: 0.15, 0.58) relative to infants receiving no breast milk. Reductions in maternal diarrhea may result in substantial reductions in diarrhea morbidity among HEU children, in addition to standard diarrhea prevention interventions.

## INTRODUCTION

Diarrhea remains a significant cause of morbidity and mortality among children living in sub-Saharan Africa (SSA), contributing to nearly 10% of under-5 deaths in the region.^1,2^ Increasing evidence suggests diarrhea, particularly moderate-to-severe diarrhea (MSD) and prolonged/persistent diarrhea, is associated with considerable long-term morbidity, including growth compromise, increased frequency of other infections, and poor cognitive development.^3–6^

In 2018, there were more than two million young women (15–24 years) in SSA living with HIV.^7^ Widespread implementation of programs to prevent mother-to-child transmission (PMTCT) of HIV has successfully reduced the risk of HIV transmission to children, resulting in a growing population of children exposed to HIV but uninfected (HEU).^8^ Being born to or living with an HIV-infected mother may present unique risk factors for diarrhea, such as frequent bouts of maternal diarrhea and/or increased maternal household antibiotic use. With 1.2 million HEU infants born each year, a reduction in diarrhea among this uniquely vulnerable population would contribute to the global decline in diarrhea burden.^9^ We determined incidence and risk factors for diarrhea, MSD, and prolonged/persistent diarrhea in a cohort of HEU infants.

## METHODS

### Study design.

This analysis used a historic cohort of HIV-infected mothers and their infants. The parent study enrolled HIV-positive pregnant women with gestation ≥ 28 weeks who attended the Kenyatta National Hospital in Nairobi from 1999 to 2001. Further details regarding this cohort have been previously described.^10,11^ During pregnancy, women were counseled on infant feeding and the risk of HIV transmission through breast milk before electing to breastfeed or formula feed. Mothers choosing to breastfeed were encouraged to exclusively breastfeed their infant for the first 4–6 months. Consistent with the national guidelines at the time, participants received short-course zidovudine for PMTCT of HIV and women with severe immunosuppression (CD4 count < 200 cells/µL) were provided with co-trimoxazole prophylaxis and referred to HIV treatment programs. Infants did not receive additional prophylaxis nor did mothers receive antiretroviral therapy (ART) during breastfeeding.

The eligibility criteria for our analysis included singleton birth or firstborn twin and at least one negative HIV polymerase chain reaction (PCR) test at a recorded study follow-up visit. Infants testing positive for HIV by 1 month of age were considered perinatally infected and excluded from the analysis.

### Data collection.

At enrollment (about 32-week gestational age), sociodemographic and pregnancy medical history were collected using a standardized questionnaire, and blood samples were collected and processed for CD4 and HIV RNA viral load (VL) determination. After delivery, mothers were observed in the clinic with their infants at 2 weeks, 4 weeks, and then monthly up to 12 months (study visits). In addition, mothers were encouraged to return to the clinic if the child was sick, from which morbidity diagnoses were recorded (sick visits). Infant morbidity, breastfeeding, and HIV status were assessed by questionnaires and clinical examinations at study visits. Maternal anthropometric measurements, CD4 count, and VL were collected at months 1, 3, 6, 9, and 12. Mothers were assessed for intercurrent illness at all study visits.

### Statistical analysis.

#### Definitions.

Infant diarrhea was defined as maternal report since the last study visit or clinician diagnosis at any study or sick visit. Clinician diagnosis of diarrhea at sick visits was considered even if diarrhea was not the primary diagnosis. Reports of diarrhea within 14 days of a previous report were counted as the same episode to avoid double counting episodes. Moderate-to-severe diarrhea was defined as diarrhea with dehydration or dysentery, or a diarrhea-associated hospitalization.^12^ Diarrhea duration was ascertained in categories of ≥ 2 days and > 1 week. We defined prolonged/persistent diarrhea to be episodes of diarrhea > 1 week.

#### Burden.

Incidence of infant diarrhea was calculated by interval censoring at the last study visit (12 months) or at the last negative HIV test before the child was lost to follow-up, died, or had a positive HIV test, whichever came first. Interval censoring methods were used to censor person-time for missed visits and 7 days before and after a diarrhea episode. Analyses were carried out for all infant diarrhea relative to no diarrhea, as well as the subset of only MSD and only prolonged/persistent diarrhea relative to no diarrhea or mild diarrhea not meeting MSD or prolonged/persistent diarrhea definitions.

#### Correlates.

Hazard ratios (HRs) and 95% CIs for potential correlates of infant diarrhea were estimated using Andersen–Gill Cox proportional hazard models, with the Efron method for ties and robust standard errors.^13^ This model allows for repeated infant diarrhea events and time-varying factors.

Potential correlates of diarrhea were specified a priori from established and suspected risk factors.^14^ Maternal health indicators during pregnancy included low maternal CD4 count (< 200, 200–499, versus ≥ 500 cells/µL), high VL (≥ 4 log_10_), low mid-upper arm circumference (MUAC, < 23.5 cm) at enrollment, and report of antibiotic use and diarrhea. Antibiotic use and maternal diarrhea reported during the first year were also included. Maternal antibiotic use was defined as maternal report of taking any antibiotic for treatment and does not include prophylaxis co-trimoxazole. Infant factors included low birth weight (< 2,500 g) and breastfeeding at study visits (ever breastfed versus never breastfed [formula fed]; feeding status in 24 hours before study visit: exclusively breastfeeding, some breastfeeding, and no breastfeeding). Other key covariates included household crowding (≥ 2 persons/room), type of toilet (pit latrine versus flush), shared toilet (versus household), and maternal education (> primary education) measured at baseline. Maternal indicators during the first year of life (diarrhea and antibiotic use) were not collected at sick child visits, and thus, sick child visits were not included in time-varying analyses for these variables. Separate models tested the effect of time-varying maternal diarrhea and antibiotic use reported 1 month before and concurrent with infant diarrhea.

Crude and adjusted models were constructed to determine independent correlates of all infant diarrhea, MSD, and prolonged/persistent diarrhea. The following variables, in order, were considered for inclusion in the adjusted models: crowding, enrollment maternal CD4 count, and maternal MUAC. These covariates were retained in the adjusted model if the HR differed from the crude model by more than 10%. Adjusted models are presented as final models unless addition of the potential confounders did not change the crude model, in which case the crude was presented as the final model. Baseline maternal characteristics between infants lost to follow-up before the first visit and those included in the analysis were compared using Welch’s *t*-test (continuous variables) and Fischer’s exact test (binary variables). All analyses used two-sided hypothesis tests with an alpha of 0.05. Analyses were conducted in Stata 15 (StataCorp, College Station, TX).

## RESULTS

### Study population.

**Figure 1.**
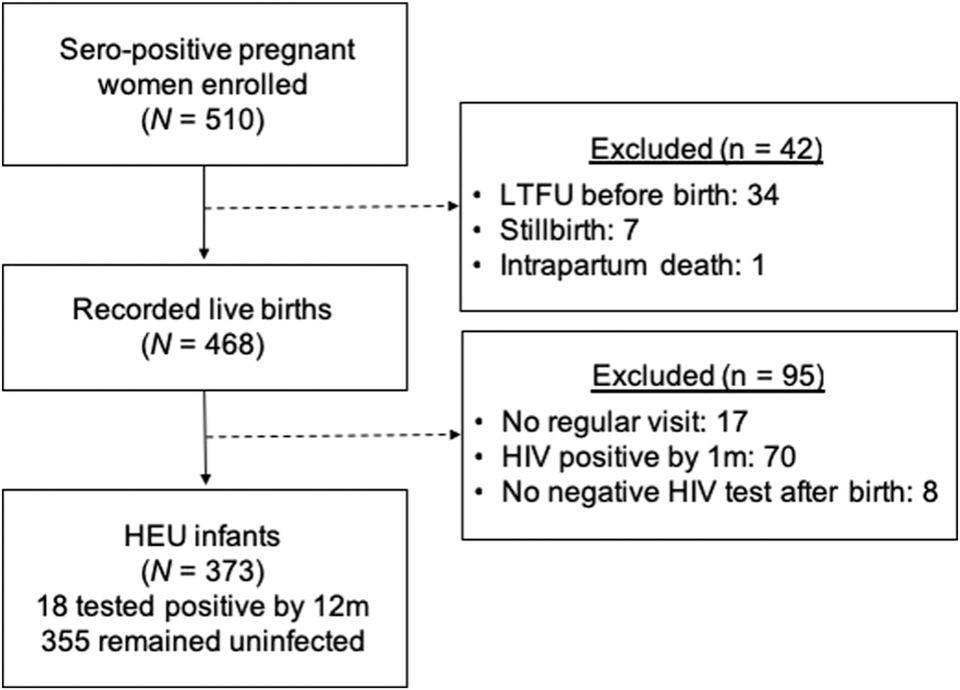
Participant flow chart.

The parent cohort enrolled 510 pregnant HIV-infected women and recorded 468 live births of singleton or firstborn twins (six second-born twins were excluded). Among the 468 infants, 70 were perinatally HIV infected, 17 were lost to follow-up before the first visit, and eight had no recorded negative HIV test at a regular study visit (Figure 1). Mothers of infants who were lost to follow-up before the first visit had a slightly higher mean log VL (difference in means = 0.42 log_10_ copies/mL; 95% CI: 0.06, 0.78) but were not statistically different in any other characteristics at baseline. The remaining 373 HEU infants were included in this analysis, including 355 who remained uninfected in the first year and 18 who subsequently acquired HIV at a median age of 181 days (interquartile range [IQR]: 90–354; Figure 1), whose visits after the last negative HIV test were censored.

At enrollment, most mother–infant pairs (84%) reported living in crowded households and used a pit latrine (52%). Mothers’ median age at enrollment was 25 years (IQR: 22–28), and 42% had more than a primary school education. Before delivery, mothers’ median CD4 count was 448 cells/µL (IQR: 316–618) and median HIV VL was 4.7 log_10_ copies/mL (IQR: 4.1–5.1); in addition, 14% were defined as undernourished (MUAC < 23.5 cm). During pregnancy, 6% mothers reported at least one episode of diarrhea and 20% reported using antibiotics. Postpartum, 22% of mothers ever reported diarrhea, among whom 127 total episodes were recorded (incidence rate: 0.40 episodes/year). The majority of women (78%) reported antibiotic use for treatment at least once for a total of 813 occasions, and 13% of women reported any co-trimoxazole prophylaxis use, indicating severe immunosuppression (Table 1).

**Table 1:** Baseline and follow-up characteristics among 373 HIV-exposed uninfected infants and their mothers*

Covariate	*N* (%)†
Total	373
Home environment factors	
Pit latrine	193 (52)
Flush toilet	180 (48)
Shared toilet	340 (91)
Household toilet	32 (9)
≥ 2 persons/room in house	315 (84)
< 2 persons/room in house	55 (15)
Maternal factors	
> Primary education	155 (42)
≤ Primary education	214 (57)
Anthropometry at 32-week gestational age	
MUAC < 23.5 cm	51 (14)
MUAC ≥ 23.5 cm	244 (65)
Maternal HIV at 32-week gestational age	
CD4 count < 200 cells/µL	28 (8)
CD4 count 200–499 cells/µL	190 (51)
CD4 count ≥ 500 cells/µL	147 (39)
log VL ≥ 4	267 (72)
log VL < 4	67 (18)
Pregnancy health	
Diarrhea	21 (6)
No diarrhea	352 (94)
Antibiotic use	75 (20)
No antibiotic use	298 (80)
Postpartum health	
Ever diarrhea	82 (22)
Never diarrhea	291 (78)
Ever antibiotic use	290 (78)
Never antibiotic use	83 (22)
Infant factors	
Birth weight < 2,500 g	21 (6)
Birth weight ≥ 2,500 g	343 (92)
Ever breastfed	279 (75)
Never breastfed	94 (25)

MUAC = mid-upper arm circumference; VL = viral load.

* Participant characteristic data also presented elsewhere (Deichsel et al.^43^).

† Percents may not add to 100% because of missing data.

### Infant diarrhea burden.

**Figure 2.**
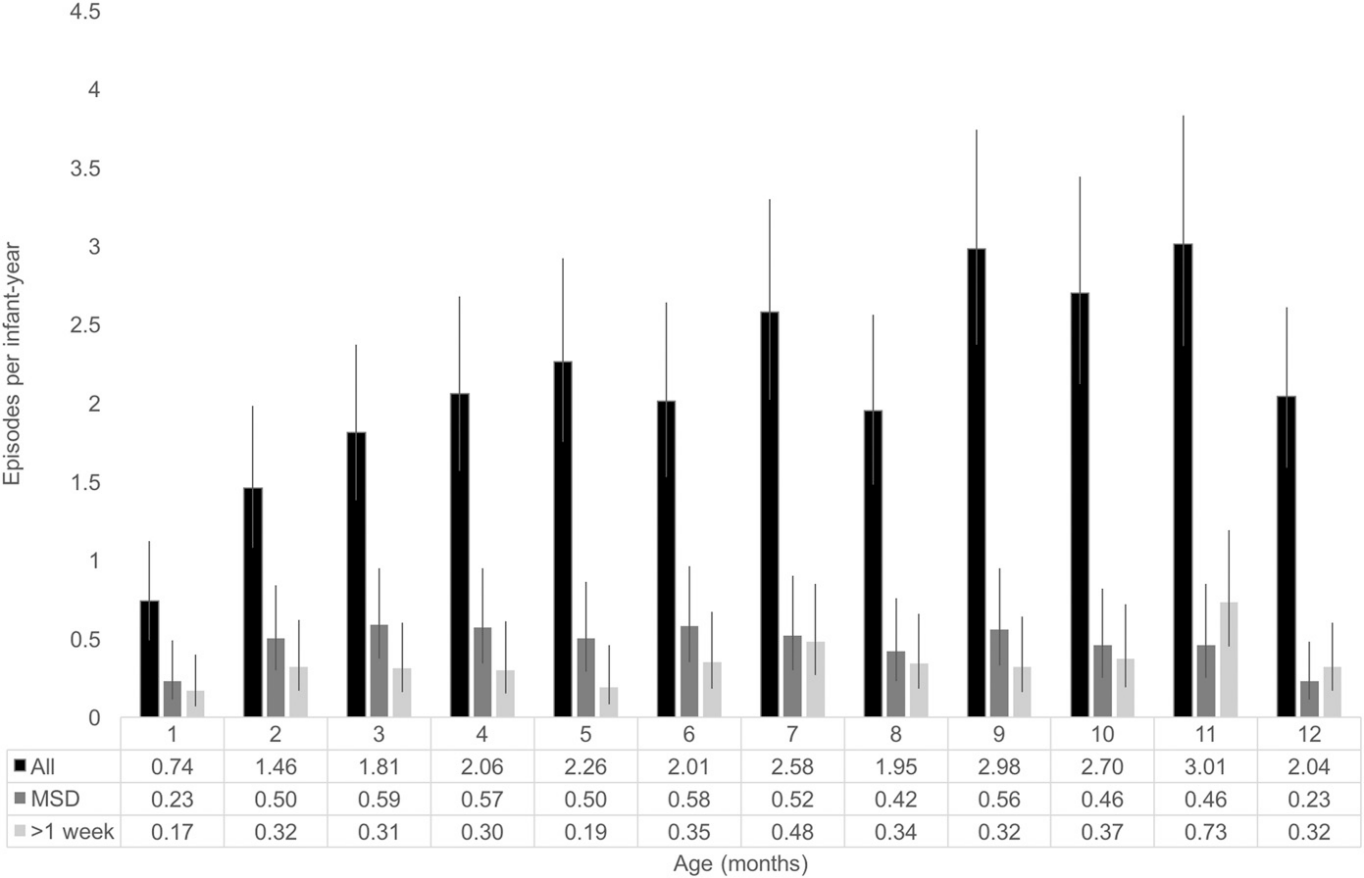
Incidence rate of all diarrhea, moderate-to-severe diarrhea (MSD), and prolonged/persistent infant diarrhea by infant age.

Enrolled infants contributed 319.3 infant-years (i-yrs) and 666 episodes of diarrhea. Of the 666 diarrhea episodes, 22% were considered MSD and 17% prolonged/persistent diarrhea (Table 2). The majority of infants (70%) experienced at least one diarrhea episode, and the cohort experienced a mean of 2.09 (95% CI: 1.93, 2.25) episodes of diarrhea, 0.47 (95% CI: 0.40, 0.55) episodes of MSD, and 0.34 (95% CI: 0.29, 0.42) episodes of prolonged/persistent diarrhea in the first year (Table 3). The incidence of all diarrhea peaked at 10 months of age when the rate of diarrhea was 3.04 per i-yr (95% CI: 2.40, 3.85). The incidence of MSD was low in the first month (0.31 episodes/i-yr; 95% CI: 0.17, 0.57), and after 2 months of age, the rate was stable at approximately 0.50 episodes/i-yr. Incidence of prolonged/persistent diarrhea fluctuated throughout the first year, with an average of about 0.33 episodes/i-yr (Figure 2).

**Table 2:** Descriptive diarrhea episodes experience by 373 HIV-exposed uninfected infants, *N* = 666

Characteristic	*n* (%)	Incidence per infant-year (95% CI)
Duration		
≥ 2 days	413 (62%)	1.29 (1.17, 1.42)
> 1 week	110 (17%)	0.34 (0.29, 0.42)
Moderate-to-severe	149 (22%)	0.47 (0.40, 0.55)
Dysentery	25 (4%)	0.08 (0.05, 0.12)
Dehydration	118 (18%)	0.37 (0.31, 0.44)
Hospitalization	16 (2%)	0.05 (0.03, 0.08)

**Table 3:** Correlates of all, moderate-to-severe, and prolonged/persistent diarrhea in HIV-exposed, uninfected infants in Kenya, *N* = 373

	All diarrhea		Moderate-to-severe diarrhea		Prolonged/persistent diarrhea	
Covariate	Incidence i-yr (95% CI)	Final HR (95% CI)	Incidence i-yr (95% CI)	Final HR (95% CI)	Incidence i-yr (95% CI)	Final HR (95% CI)
Total	2.09 (1.93, 2.25)	–	0.47 (0.40, 0.55)	–	0.34 (0.29, 0.42)	–
Home environment factors						
Pit latrine	2.44 (2.22, 2.69)	1.44 (1.19, 1.74)*	0.55 (0.45, 0.68)	**1.49 (1.04, 2.14)**	0.36 (0.28, 0.46)	1.07 (0.67, 1.73)
Flush toilet	1.69 (1.49, 1.91)	Ref	0.37 (0.28, 0.48)	Ref	0.33 (0.25, 0.44)	Ref
Shared toilet	2.15 (1.99, 2.33)	1.30 (0.88, 1.91)†	0.48 (0.40, 0.56)	1.68 (0.92, 3.04)‡	0.36 (0.29, 0.43)	1.73 (0.62, 4.81)†‡
Household toilet	1.54 (1.16, 2.04)	Ref	0.38 (0.22, 0.68)	Ref	0.26 (0.13, 0.51)	Ref
≥ Two persons/room in house	2.17 (2.00, 2.35)	**1.35 (1.04, 1.76)**	0.46 (0.39, 0.55)	1.07 (0.68, 1.68)	0.37 (0.30, 0.45)	1.38 (0.63, 3.03)‡
< Two persons/room in house	1.62 (1.30, 2.01)	Ref	0.43 (0.29, 0.66)	Ref	0.24 (0.13, 0.42)	Ref
Maternal factors						
> Primary education	1.89 (1.67, 2.14)	0.86 (0.71, 1.04)	0.42 (0.32, 0.54)	0.84 (0.58, 1.20)	0.22 (0.15, 0.31)	**0.42 (0.22, 0.80)**‡
≤ Primary education	2.19 (1.99, 2.42)	Ref	0.50 (0.40, 0.61)	Ref	0.43 (0.34, 0.54)	Ref
Anthropometry at 32-week gestational age						
MUAC < 23.5 cm	2.03 (1.64, 2.52)	0.98 (0.73, 1.30)	0.58 (0.39, 0.87)	1.23 (0.75, 2.02)	0.48 (0.31, 0.75)	1.71 (0.83, 3.51)
MUAC ≥ 23.5 cm	2.09 (1.90, 2.29)	Ref	0.47 (0.39, 0.57)	Ref	0.29 (0.22, 0.37)	Ref
Maternal HIV at 32-week gestational age						
CD4 count < 200 cells/µL	1.62 (1.16, 2.26)	0.76 (0.55, 1.07)	0.37 (0.19, 0.74)	0.79 (0.36, 1.72)	0.09 (0.02, 0.37)	0.32 (0.08, 1.27)‡
CD4 count 200–499 cells/µL	2.08 (1.88, 2.32)	0.97 (0.80, 1.18)	0.48 (0.38, 0.59)	1.01 (0.70, 1.45)	0.35 (0.27, 0.45)	0.87 (0.49, 1.55)‡
CD4 count ≥ 500 cells/µL	2.15 (1.91, 2.42)	Ref	0.47 (0.36, 0.61)	Ref	0.36 (0.27, 0.48)	Ref
log VL ≥ 4	2.04 (1.86, 2.23)	1.04 (0.82, 1.32)	0.51 (0.42, 0.61)	**1.87 (1.04, 3.33)**	0.33 (0.26, 0.41)	0.86 (0.41, 1.83)‡
log VL < 4	1.99 (1.66, 2.38)	Ref	0.27 (0.17, 0.44)	Ref	0.34 (0.22, 0.53)	Ref
Pregnancy health						
Diarrhea	2.69 (2.03, 3.56)	1.33 (0.99, 1.79)	0.60 (0.33, 1.09)	1.33 (0.84, 2.11)	0.49 (0.26, 0.95)	1.28 (0.56, 2.94)‡
No reported diarrhea	2.05 (1.89, 2.22)	Ref	0.46 (0.39, 0.54)	Ref	0.34 (0.28, 0.41)	Ref
Antibiotic use	2.15 (1.82, 2.53)	1.06 (0.84, 1.33)	0.51 (0.36, 0.71)	1.11 (0.71, 1.74)	0.34 (0.22, 0.51)	1.00 (0.55, 1.83)‡§
No reported antibiotic use	2.07 (1.90, 2.25)	Ref	0.46 (0.38, 0.55)	Ref	0.35 (0.28, 0.43)	Ref
Postpartum health at study visit						
Diarrhea	4.41 (3.21, 6.06)	2.09 (1.43, 3.06)*	0.70 (0.31, 1.55)	**2.89 (1.10, 7.59)**‖	0.58 (0.24, 1.40)	0.83 (0.21, 3.26)‡
No reported diarrhea	1.86 (1.71, 2.03)	Ref	0.24 (0.19, 0.30)	Ref	0.32 (0.26, 0.40)	Ref
Antibiotic use	2.09 (1.75, 2.48)	1.09 (0.87, 1.36)	0.08 (0.03, 0.20)	0.46 (0.14, 1.53)§	0.46 (0.32, 0.67)	**1.63 (1.04, 2.55)**
No reported antibiotic use	1.82 (1.65, 2.01)	Ref	0.18 (0.13, 0.25)	Ref	0.27 (0.21, 0.35)	Ref
Infant factors						
Birth weight < 2,500 g	2.30 (1.66, 3.19)	1.13 (0.74, 1.71)	0.58 (0.30, 1.11)	1.40 (0.65, 3.04)‖	0.51 (0.26, 1.02)	0.86 (0.35, 2.09)§
Birth weight ≥ 2,500 g	2.07 (1.92, 2.24)	Ref	0.46 (0.39, 0.55)	Ref	0.34 (0.28, 0.41)	Ref
Breastfed	2.09 (1.92, 2.29)	1.02 (0.81, 1.28)	0.42 (0.34, 0.51)	0.69 (0.47, 1.00)¶	0.38 (0.31, 0.47)	1.41 (0.65, 3.05)‡
Formula fed	2.07 (1.78, 2.40)	Ref	0.61 (0.46, 0.80)	Ref	0.24 (0.16, 0.38)	Ref
Exclusive breastfeeding	1.06 (0.83, 1.36)	0.71 (0.49, 1.01)	0.20 (0.11, 0.35)	0.30 (0.15, 0.58)*	0.26 (0.16, 0.43)	1.33 (0.45, 3.93)§
Some breastfeeding	2.35 (2.08, 2.65)	1.05 (0.87, 1.28)	0.43 (0.32, 0.57)	0.72 (0.48, 1.06)	0.38 (0.28, 0.51)	1.29 (0.66. 2.53)§
No breastfeeding	2.32 (2.09, 2.59)	Ref	0.60 (0.49, 0.74)	Ref	0.36 (0.27, 0.47)	Ref

HR = hazard ratio; i-yr = infant year; MUAC = mid-upper arm circumference; VL = viral load. Values in bold have *P*-value < 0.05.

* *P-*value < 0.001.

† Adjusted for crowding.

§ Adjusted for 32-week gestation CD4 percent.

‡ Adjusted for 32-week maternal undernutrition (MUAC < 23.5 cm).

‖ Adjusted for all 3.

¶ *P*-value = 0.051.

### All diarrhea.

The household environment was associated with infant diarrhea. Infants living in households with a pit latrine or in crowded homes had almost 1.5 times higher risk of diarrhea than infants with a flush toilet or non-crowded households (HR: 1.44; 95% CI: 1.19, 1.74; HR: 1.35; 95% CI: 1.04, 1.76, respectively, Table 3). Shared versus household toilet was not significantly associated with infant diarrhea.

Maternal diarrhea during follow-up was associated with an approximately 2-fold increase in infant diarrhea (HR: 2.09; 95% CI: 1.43, 3.06). Other maternal indicators, such as more than a primary education, maternal CD4, VL, or antibiotic use during pregnancy, were not associated with infant diarrhea.

### Moderate-to-severe diarrhea.

Moderate-to-severe diarrhea was associated with toilet type. Infants living in a home with a pit latrine had a 49% increased risk for MSD (HR: 1.49; 95% CI: 1.04, 2.14) relative to those with a flush toilet, and children with a shared toilet had a 68% increased risk for MSD compared with those with a household toilet, with a trend for an association (HR: 1.68; 95% CI: 0.92, 3.04).

Maternal diarrhea during follow-up was associated with a 3-fold increase in infant MSD (HR: 2.89; 95% CI: 1.10, 7.59). Higher maternal VL at enrollment was associated with an increased risk of infant MSD (HR: 1.87; 95% CI: 1.04, 3.33), but maternal CD4 count was not (< 200 cells/µL; HR: 0.79; 95% CI: 0.36, 1.72 and 200–499 cells/µL; HR: 1.01; 95% CI: 0.70, 1.45).

Infants currently being exclusively breastfed had a 70% decreased risk of MSD (HR: 0.30; 95% CI: 0.15, 0.58), and infants who had ever been breastfed had a 31% decreased risk of MSD compared with those who never received breast milk, with a trend for association (HR: 0.69; 95% CI: 0.47, 1.00; *P*-value = 0.051).

### Prolonged/persistent diarrhea.

In contrast to all diarrhea and MSD, prolonged/persistent diarrhea was not associated with household environment factors. However, infants born to mothers with more than a primary education had a 58% decreased risk of prolonged/persistent diarrhea (HR: 0.42; 95% CI: 0.22, 0.80). Infants with mothers reporting antibiotic use had a 63% increased risk for prolonged/persistent diarrhea (HR: 1.63; 95% CI: 1.04, 2.55).

## DISCUSSION

In this cohort of HEU infants, diarrhea, including MSD and prolonged/persistent diarrhea, occurred frequently during the first year of life. Risk factors varied by type of diarrhea with any diarrhea primarily associated with likelihood of infectious exposure (toilet type, crowding, and maternal diarrhea), whereas risk factors for MSD included likelihood of an infectious exposure (toilet type and maternal diarrhea) and factors potentially associated with a child’s ability to fight the infection (high maternal VL and breastfeeding). Prolonged/persistent diarrhea was linked to low socioeconomic status (maternal education) and maternal antibiotic use, a potential indicator of mother’s overall health.

Diarrhea incidence in this HEU cohort was slightly lower than the 3.5 episodes/child-year incidence reported from children in Nairobi during this period.^15^ The lower rates of infant diarrhea in our study relative to other contemporaneously published estimates may be influenced by ascertainment of diarrhea at monthly clinic visit rather than more frequent home visits used in other studies. Because of longer periods between data collection, mothers may have only reported more severe diarrhea episodes.^16^ Our study documented a peak in infant diarrhea incidence around 9–11 months of age, compatible with the peak incidence (6–9 months of age) previously reported in HEU^17,18^ and other cohorts.^19^

Our cohort had similar rates of prolonged/persistent diarrhea and MSD as recent studies,^3,15^ including a nearly identical incidence rate of MSD to that reported from western Kenya, a region of high maternal HIV prevalence, with 0.51 episodes/i-yr.^12^ Although diarrhea burden is generally thought to be declining in the SSA, the rate of decline of the more severe forms of diarrhea may be stalled in populations with a number of HEU or other vulnerable children.

We evaluated risk factors for three types of diarrhea to elucidate overlapping and distinct mechanisms. There are limited data comparing risk factors between all reported diarrhea and more severe types of diarrhea (MSD and prolonged/persistent) associated with long-term sequelae, particularly among HEU infants.^12,20^

We found that the risk factors for any diarrhea were linked to the likelihood that a child was exposed to an infectious agent through crowding, toilet type, and maternal diarrhea. Flush toilets were associated with a reduced risk of diarrhea, supporting the inclusion of improved sanitation components in diarrhea prevention strategies even for children not yet old enough to use the toilets themselves.^21,22^ Toilet type in our cohort may also represent lower socioeconomic status and limited access to clean water or other risk factors for diarrhea. Holistic approaches to families with HIV, including addressing water and sanitation, are likely to yield improved health.^23^

Our finding of an association between maternal diarrhea and infant diarrhea is consistent with Zambia and Malawi studies among HEU children.^24,25^ The association between maternal diarrhea and infant diarrhea and MSD is likely due to the shared environment or person-to-person pathogen transmission. Maternal–infant illnesses are often shared and underscore the importance of concurrently addressing mother and child health to optimize child growth and developments. Interventions in caregivers likely impact child health as evidenced by reductions in infant diarrhea associated with maternal ART and multivitamins.^18,26,27^

Similar to all diarrhea, MSD was associated with household and environmental factors (pit latrine and maternal diarrhea). In addition, exclusive breastfeeding and low maternal VL were protective against MSD, suggesting that these factors influenced the infant’s capacity to contain diarrheal infection. International guidelines recommend exclusive breastfeeding for all infants in the first 6 months of life as a key diarrhea prevention strategy.^28,29^ It is possible that breastfeeding prevents exposure to or protects against pathogens causing more severe diarrhea.^30,31^ The increased risk of MSD among infants of mothers with high VL may stem from impaired infant immune responses following in utero exposure to maternal HIV.^32–34^ Although today there is higher coverage of ART for mothers, resulting in lower VLs, with an estimated 80% of HIV-infected mothers globally being virally suppressed during pregnancy. These data again highlight the importance of a strong maternal immune system for her infants’ health.^35^

In contrast to all diarrhea and MSD, prolonged/persistent diarrhea was associated with lower maternal education and postpartum maternal antibiotic use. Low maternal education is an established predictor for poor childhood health outcomes, including diarrhea^3,36^ and malnutrition,^37^ the latter being an established consequence of prolonged/persistent diarrhea.^3^ Antibiotics taken by the mother may be transferred to the infant through breastfeeding, possibly disrupting the infant’s microbiome and increasing the risk of antibiotic-associated diarrhea.^38^ However, such a reason would not explain maternal antibiotic’s unique association with prolonged/persistent diarrhea rather than all diarrhea or MSD. It may be more likely that maternal antibiotic use during the postpartum period is an indicator of declining maternal health and immunity which predisposes to infections and risk of infant persistent diarrhea. Given the infant immune system is especially implicated in prolonged/persistent diarrhea, it could be that the association between maternal antibiotic use and infant prolonged/persistent diarrhea is explained by a parallel decline in both mother and child health. There is increasing evidence suggesting exposure to maternal antibiotic in utero may affect infant microbiome and immune development, potentially increasing susceptibility and severity of childhood infections.^39–41^ However, although we found an association with postpartum maternal antibiotic use and infant prolonged/persistent diarrhea, we did not find associations with prepartum maternal antibiotic use.

There are several limitations of this historic cohort study. The data were collected before the widespread maternal ART use, HEU co-trimoxazole prophylaxis, and childhood rotavirus vaccination, three interventions that may reduce infant diarrhea. However, this historic cohort provides a unique opportunity to define how maternal diarrhea contributes to infant diarrhea in the absence of such interventions, thereby providing a natural history. Also, maternal diarrhea was common in this cohort likely because of the lack of these interventions, increasing the power to detect mother/infant associations. Maternal CD4 count was not associated with infant diarrhea in the present cohort, and associations between maternal morbidity and infant diarrhea may persist, despite ART-associated improvements in maternal immunity. Second, the cohort was not initially designed to focus on diarrhea, and diarrhea may have been under ascertained because reports were collected monthly. We accepted diagnosis or report of diarrhea even if it was not the primary diagnosis for a sick child visit, likely capturing some diarrhea that was secondary to other illness. The study may also suffer from nonrandom loss to follow-up. For example, formula-fed infants were more likely to die, and to do so early in follow-up, than breastfeeding infants in this cohort. This may partially explain the failure to detect a protective relationship between breastfeeding and all infant diarrhea.^30,42^

## CONCLUSION

In summary, we found household and environmental factors both predicted all diarrhea and MSD, while exclusive breastfeeding and low maternal VL were uniquely protective for MSD, and maternal education and postpartum maternal antibiotic use appeared important for prolonged/persistent diarrhea. Differing risk factors between all diarrhea and more severe types of infant diarrhea may represent distinct mechanisms for these diarrhea pathologies. Identification of maternal diarrhea and antibiotic use may be an opportunity to identify high-risk infants and deliver interventions to prevent or treat infant diarrhea. Targeted delivery of interventions to HIV-infected caregivers during regular clinic visits, including education on signs of severe child illness, encouraging care-seeking behavior, breastfeeding support, and oral rehydration salts and zinc, may result in substantial reductions in diarrhea morbidity and mortality among HEU infants.
